# Improving ST-Elevation Myocardial Infarction (STEMI) Care Through the Bahrain Stent Save a Life (BaSSaL) Program: A Prospective Study

**DOI:** 10.7759/cureus.92091

**Published:** 2025-09-11

**Authors:** Haitham Amin, Husam Noor, Fuad Abdulqader, Sadananda Shivappa, Shereen M Alshaikh, Fawaz Bardooli, Tajammul Hussain, Ali J Alhashli, Najla M Alnetaifi, Tarique S Chachar, Nouf AlAnsari, Abdulrahman Masood, Nooraldaem Yousif

**Affiliations:** 1 Interventional Cardiology, Mohammed Bin Khalifa Bin Salman Al Khalifa Specialist Cardiac Centre, Awali, BHR; 2 Cardiology, Mohammed Bin Khalifa Bin Salman Al Khalifa Specialist Cardiac Centre, Awali, BHR

**Keywords:** bahrain, myocardial infarction, primary pci, registry data, st-elevation myocardial infarction (stemi)

## Abstract

Aim: This study aimed to characterize the clinical features and predictors of adverse cardiovascular events in patients with ST-elevation myocardial infarction (STEMI) in Bahrain.

Methods: Patients who presented with STEMI between January 2022 and December 2022 and underwent primary percutaneous coronary intervention (PPCI) are included in this single-center prospective study. We monitored each patient for a year after their initial hospitalization.

Results: Of the 787 patients in our study, 487 (62%) were not Bahraini. The majority of participants, 720 (91.5%), were men. The mean age was 56.6±10.88 years. Stent thrombosis occurred in eight patients (1.0%). During their hospitalization, 33 patients (4.2%) died. Fifty-three patients (6.7%) died within a year, including 20 patients (2.5%) who died during the follow-up after being discharged from the hospital.

Conclusion: The establishment of nationwide quality control registries of successive STEMI patients, similar to the one we have with the Bahrain Stent Save a Life (BaSSaL) project, aids in pinpointing particular barriers to the implementation of guidelines and defining actions to guarantee that most STEMI patients may have lifesaving PPCI. Improving patient access to PPCI will reduce total ischemic time and mortality and help meet target revascularization timelines.

## Introduction

Cardiovascular disease (CVD) is the leading cause of death in Bahrain. Between 2009 and 2019, the number of CVD-related deaths increased by 50% [1]. Acute coronary syndromes (ACS) are mostly caused by ST-segment elevation myocardial infarction (STEMI), which requires prompt diagnosis and treatment to reduce mortality, disability, and sequelae. Primary percutaneous coronary intervention (PPCI) is the gold standard for treating STEMIs and is recommended as the first-line treatment if available in PCI-capable centers that can offer round-the-clock coverage and within an acceptable time frame (first medical contact, or FMC, to a device time of less than 120 min). All the international guidelines endorse this approach [2].

The Bahrain Stent Save a Life program, launched on January 1, 2022, is a nationwide PPCI initiative in the Kingdom that aims to lower mortality and disability associated with cardiac diseases [1]. Its objective is to provide all patients with STEMI nationwide with prompt and accurate diagnosis and treatment. Despite these alarming trends and illness burden, current data on risk factors, clinical characteristics, practice patterns, and outcomes among patients presenting with STEMI in Bahrain are lacking. Large-scale statistics from Gulf registries that have been previously published may not be fully representative of Bahrain’s unique demographics. Most cases are young, male, and non-Bahraini patients with lower socioeconomic status [3,4].

A prospective study of STEMI in the population of Bahrain may highlight areas of weakness in the current practice paradigm that may be addressed to improve STEMI outcomes, besides identifying common risk factors that may be modified to lower the risk of coronary artery disease. The present study aimed to prospectively and comprehensively define the clinical characteristics, evidence-based therapy, risk factors, clinical outcomes, and predictors of adverse cardiovascular events in patients in Bahrain who present with STEMI.

## Materials and methods

Setting

This is a single-center prospective study that includes patients who presented with ST-elevation myocardial infarction (STEMI) between January 2022 and December 2022 and underwent primary percutaneous coronary intervention (PPCI) at Mohammed Bin Khalifa Bin Salman Al Khalifa Specialist Cardiac Centre, the only tertiary governmental cardiac center in Bahrain, where all PPCIs are performed.

Eligibility

Patients who meet the European Society of Cardiology (ESC) recommendations for STEMI diagnosis include those with persistent ST-segment elevation on the electrocardiogram (ECG) with acute chest pain (or chest pain-equivalent signs/symptoms) [5]. Moreover, STEMI equivalents, such as hyperacute T waves, left bundle branch block (LBBB), or paced rhythm with Sgarbossa criteria, de Winter's sign, and posterior STEMI, were added. Furthermore, PPCI was used to treat high-risk ECGs, including Wellen’s ECG and ST-Elevation in Augmented lead on ECG (STE aVR), with multi-lead ST depression in leads I, II, aVF, and V4-V6. The case was excluded and labeled as a STEMI mimic if the angiography showed no obstructive disease that might explain the presentation. Patients who showed up more than 72 hours after the symptom onset were not included in this study.

Data collection

A detailed data collection form and study protocol were approved by the Research Ethics Committee (IRB #CTD-RES-2023-0045), and written informed consent was obtained from all eligible study participants. In post-arrest or intubated patients, the next of kin or the immediate first-degree relative provided consent.

Definitions

Risk factors were defined using ESC guidelines and recommendations. Hypertension was defined as a systolic blood pressure of ≥140 mmHg, a diastolic blood pressure of ≥90 mmHg, or treatment for hypertension. Dyslipidemia is defined as any lipid abnormality that includes total cholesterol ≥240 mg/dL, low-density lipoprotein (LDL) ≥160 mg/dL, serum triglycerides ≥150 mg/dL, high‐density lipoprotein-cholesterol <40 mg/dL in men or <50 mg/dL in women, or treatment of dyslipidemia. Diabetes mellitus (DM) was defined as fasting glucose ≥7.0 mmol/L (≥126 mg/dL), HbA1c level ≥48 mmol/mol (≥6.5%), or treatment for DM. Chronic kidney disease is defined as an estimated glomerular filtration rate of less than 60 mL/min/m^2^, a diagnosis of chronic kidney disease, or dialysis. Peripheral artery disease is defined as a history of peripheral artery revascularization, an ankle-brachial index <0.9, or lower-extremity artery stenosis ≥50%. A history of smoking was defined as current (tobacco products used within the last month), former, or never.

Outcomes

Endpoints at the one-year follow-up comprised all-cause death. Other outcomes studied were in-hospital mortality and target time (first medical contact {FMC}-to-device time) for PPCI. The ESC 2017 STEMI guidelines were used as a benchmark to align with international standards at every stage of the patient journey - from initial presentation at a medical facility to the performance of PPCI - covering onset-to-first medical contact (FMC), FMC-to-device time, and total ischemic time.

Statistical analysis

Continuous variables are represented as mean±standard deviation or median (interquartile range), whereas categorical variables are presented as frequencies and percentages (n {%}). Normality was assessed using the Shapiro-Wilk test. We used the statistical software package SPSS 24 (Armonk, NY: IBM Corp.) for Windows to perform statistical analysis and produce graphs and plots.

Follow-up

All study participants were followed up for one year after the index hospitalization via in-clinic follow-up and telephone calls. The study team recorded vital signs, adherence to guideline-directed medical therapy, target goals for secondary prevention, and the occurrence of cardiovascular events during follow-up.

## Results

Demographics

A total of 787 patients were included, with 62% (n=487) being non-Bahraini. Overall, 720 (91.5%) patients were men. Male dominance was constant regardless of nationality but was more obvious in non-Bahraini patients than in Bahraini patients - 462 men (97.5%) vs. 258 men (82.4%), respectively. The mean age was 56.6±10.882 years. The mean age of non-Bahraini patients was lower than that of Bahraini patients (49±9 vs. 59±12 years). An independent t-test confirmed that non-Bahraini patients were significantly younger than Bahraini patients (p<0.001) (Table 1).

**Table 1:** Baseline characteristics of 787 patients enrolled in the BaSSaL registry. STEMI: ST-elevation myocardial infarction; DM: diabetes mellitus; HTN: hypertension; CKD: chronic kidney disease; CVA: cerebrovascular accident; PCI: percutaneous intervention; CABG: coronary artery bypass grafting; WBC: white blood cell; BaSSaL: Bahrain Stent Save a Life; LDL: low-density lipoprotein; PDV: peripheral vascular disease

Variables	Values
Age (years)	
General population (mean±SD)	56.6±10.882
Bahraini (mean±SD)	59±12
Non-Bahraini (mean±SD)	49±9
Gender	
Male, n (%)	720 (91.5)
Female, n (%)	67 (8.5)
Bahraini (n=313)	
Male, n (%)	258 (82.4)
Female, n (%)	55 (17.6)
Non-Bahraini (n=474)	
Male, n (%)	462 (97.5)
Female, n (%)	12 (2.5)
Nationality	
Bahraini, n (%)	313 (39.8)
Non-Bahraini, n (%)	474 (60.2)
Bahraini (female), n (%)	55 (17.6)
Non-Bahraini (female), n (%)	12 (2.5)
STEMI, n (%)	776 (98.6)
STEMI equivalent, n (%)	6 (0.8)
STEMI mimics, n (%)	5 (0.6)
Location of MI	
Anterior, n (%)	389 (50.1)
Inferior, n (%)	343 (44.2)
Posterior, n (%)	19 (2.5)
Lateral, n (%)	25 (3.2)
Cardiovascular risk factors	
DM, n (%)	334 (42.8)
HTN, n (%)	346 (44.4)
Dyslipidemia, n (%)	236 (30.3)
Smoker (current), n (%)	268 (34.4)
Smoker (former), n (%)	34 (4.4)
CKD, n (%)	15 (1.9)
Dialysis, n (%)	6 (0.8)
CVA, n (%)	11 (1.4)
PDV, n (%)	3 (0.4)
Previous PCI, n (%)	64 (8.2)
Previous CABG, n (%)	4 (0.5)
Laboratory results	
Mean hemoglobin (mean±SD)	14.01±1.95
Anemia (HB <11 g/dL), n (%)	54 (6.9)
Mean WBC (mean±SD)	11.52±4.16
Leukocytosis (WBC ≥11×10^9^/L), n (%)	397 (51.0)
Mean HbA1c in general population (mean±SD)	7.34±2.28
Mean HbA1c in known DM (mean±SD)	8.86±2.32
New DM, n (%)	84 (19.0)
Mean LDL in general population (mean±SD)	3.5±1.07
Mean LDL in known dyslipidemia (mean±SD)	3.39±1.13
New dyslipidemia, n (%)	735 (95.0)
Triglycerides (mean±SD)	1.44±1.16
HDL (mean±SD)	1.06±0.27
Ejection fraction	
≤40, n (%)	373 (48.8)
41-49, n (%)	186 (24.4)
≥50, n (%)	205 (26.8)

STEMI types

Among the 776 cases identified as true STEMI (98.6%), 389 (50.1%) were anterior, 343 (44.2%) were inferior, 19 (2.5%) were posterior, and 25 (3.2%) were lateral. The catheterization laboratory was falsely activated in five cases (0.6%). Although anterior STEMI (50.1%) was more frequent than inferior STEMI (44.2%), the difference did not reach statistical significance (χ² test, p=0.08) (Table 1).

Laboratory investigations and cardiovascular risk factors

The predominant risk factor detected during the index procedure was dyslipidemia, present in 735 (95%) patients, followed by newly diagnosed diabetes mellitus in 149 (19%) individuals. Diabetes was observed in 337 patients (42.8%) and systemic hypertension in 350 patients (44.4%), establishing them as the predominant cardiovascular risk factors in the medical history (Table 1).

Procedural characteristics

In our practice of PPCI, GPIIb/IIIa inhibitors were used in 198 patients (25.2%), thrombectomy was performed in 241 patients (30.6%), and intravascular imaging was employed in 84 patients (10.7%). Saphenous venous graft-percutaneous intervention (SVG-PCI) was infrequently identified as a cause of STEMI, occurring in merely three patients, representing 0.4% of cases. Stent thrombosis was observed in eight (1.0%) patients. Radial access succeeded in 728 patients (92.6%), whereas conversion to a femoral approach was required in 15 cases (1.9%). The median time from FMC to device use was 123 min in patients referred from other healthcare facilities (HCF). Almost half of the patients were discharged with reduced left ventricular systolic function (LVSF) (ejection fraction {EF} <40%) (Table 2).

**Table 2:** Procedural characteristics of primary PCI for 787 patients enrolled in the BaSSaL registry. IABP: intra-aortic balloon pump; GP2B3A: glycoprotein 2B3A; CABG: coronary artery bypass grafting; SVG: saphenous venous graft; PCI: percutaneous coronary intervention; FMC: first medical contact; POBA: plain old balloon angioplasty; IVUS: intravascular ultrasound; LM: left main; ISR: in-stent restenosis; SVG: saphenous venous graft; PCI: percutaneous coronary; OCT: optical coherence tomography; CAD: coronary artery disease; BaSSaL: Bahrain Stent Save a Life

Variables		n (%)
Inotropes+IABP		37 (4.7%)
GP2B3A		198 (25.2%)
Thrombectomy		240 (30.6%)
POBA only		31 (3.9%)
Direct stent		225 (28.7%)
Number of stents		
0		116 (14.8%)
1		577 (73.5%)
2		86 (11.0%)
3		6 (0.8%)
Diagnostic angiogram (no PCI)		69 (8.8%)
Emergency/urgent CABG		2 (2.9%)
SVG-PCI		3 (0.4%)
LM-PCI		22 (2.8%)
Intravascular imaging		
IVUS	55 (7.0%)	
OCT	29 (3.7%)	
Severity of CAD		
2 vessels CAD	191 (24.3%)	
3 vessels CAD	137 (17.5%)	
Staged PCI		
Staged PCI-index admission	29 (3.7%)	
Staged PCI-OPD	35 (4.5%)	
Stent failure		
Stent thrombosis	8 (1.0%)	
ISR	7 (0.9%)	
Vascular access		
Radial	722 (92.6%)	
Femoral	43 (5.5%)	
Radial/femoral	15 (1.9%)	
Emergency medical services (EMS) transfers		
Time	Median (min)	
Onset of symptoms to FMC	52	
FMC to device	88	
Healthcare facilities (HCF) transfers		
Time	Median (min)	
Onset of symptoms to FMC	120	
FMC to device time	123	

Emergency medical services (EMS) utilization and pre-hospital activation

The national ambulance EMS paramedics were enrolled in training programs before the start of the BaSSaL program to help in the recognition of STEMI symptoms, instructed on how to perform a 12-lead ECG in the field, and successfully transmit the ECG to the STEMI Hotline. "Calling 999 for acute chest pain" was a media campaign that was started in parallel with starting the STEMI program to help encourage more use of EMS services and prevent patients with chest pain from driving to healthcare facilities (HCF). Of the total cohort of STEMI patients, only 9% (68 cases) were referred to EMS, and they were directly transferred from the field to our center. These patients with pre-hospital activation had shorter FMC-to-device median times (88 min) compared to those who presented with other HCFs (including secondary care hospitals, primary care centers, and private hospitals and clinics) and had to be transferred to our PPCI center (FMC-to-device median time: 123 min).

Mortality

During the hospital stay for PPCI, 33 (4.2%) patients died. An additional 20 patients (2.5%) died post-discharge, resulting in a one-year mortality of 53 patients (6.7%) (Table 3).

**Table 3:** In-hospital and 1-year mortality for 787 patients enrolled in the BaSSaL registry. BaSSaL: Bahrain Stent Save a Life

In-hospital mortality	33 (4.2%)
1-year mortality	53 (6.7%)

## Discussion

A comprehensive registry is required to understand the disease burden and reflect the current state of STEMI in Bahrain. Additionally, it complements the findings of previous registries of ACS in the Gulf, including the Saudi Acute Myocardial Infarction Registry (STARS), which enrolled 2,233 patients (1,471 with STEMI) from May 2015 to January 2017; the Gulf COAST registry, which studied 1,039 patients with STEMI from four Gulf countries (Bahrain, Kuwait, Oman, and the United Arab Emirates) from January 2012 to January 2013; and the Second Gulf Registry of Acute Coronary Events (Gulf RACE-II), which was a multinational observational study of 4,723 ACS (STEMI/NSTE-ACS) from October 2008 to June 2009. These registries have consistently shown an increase in the incidence of STEMI in our region, notable delays in receiving PPCI, a high percentage of patients receiving thrombolysis as their primary reperfusion strategy due to limited access to PPCI-capable centers, and underutilization of evidence-based medical therapies [3,4,6].

The distinctive strengths of the BaSSaL registry will enhance our understanding of STEMI in Bahrain. First, this research provides a current perspective on the epidemiology and treatment of STEMI in Bahrain. There is limited information available on individuals hospitalized in Bahrain due to STEMI over the past few years. Given the substantial scientific advancements in STEMI management over the past 10 years, as evidenced by the ESC STEMI guidelines 2017 and ESC ACS guidelines 2023, as well as the ongoing rapid evolution of risk factor profiles in Bahrain, research on a population representative of current practice must be conducted. Second, this was a single-center experience with a high patient volume treated in Bahrain’s only full-service catheterization laboratories and the only medical team and institution offering PPCI with nearly 100% follow-up. Furthermore, all national and foreign nationals would be eligible for this program, which would maximize the utilization of resources available for healthcare and yield findings that could be applied to the entire population.

The goal of the ESC’s “stent for life” campaign is to raise the proportion of patients receiving PPCI across Europe, the Mediterranean region, and other parts of the world. Based on the ACVC-EAPCI EORP STEMI Registry of the ESC, PPCI was performed in 80% of patients in ESC member countries in Europe and 75% in ESC member countries in the Middle East. In North Africa and non-ECS Middle Eastern countries, the rate is only approximately 50% [7]. In the BaSSaL registry, PPCI was performed in 91.2% of the patients; the remaining patients were either medically treated or underwent emergent CABG. Owing to the establishment of STEMI networks with clear patient pathways through Bahrain’s statewide PPCI program, patients now have more access to this life-saving procedure. As expected, patients presenting with STEMI in Bahrain had reduced rates of morbidity and mortality.

PPCI is the preferred reperfusion strategy for STEMI, provided that it can be performed expeditiously (i.e., 120 min from the STEMI diagnosis) by an experienced team. High-volume centers can perform PPCI faster and result in lower mortality, according to real-life data [5]. As demonstrated, in the first year of the BaSSaL program, the median time from FMC to device was 123 (102-159) min, which is likely to improve in the following years. Notably, the patient’s related delay, the 120 (53-300) min period between the start of symptoms and FMC, contributes significantly to the increased total ischemic time. This delay is deemed “high range” in comparison to patient-related delays documented in other international studies.

The use of EMS with pre-hospital activation was only 9% in our registry. Although these numbers are low, they are higher than the 4% reported in previous Gulf studies [8]. EMS use was associated with shorter FMC-to-device time compared to patients who presented with HCF by themselves (88 vs. 123 min). This cohort also had a shorter onset of symptoms at FMC (52 vs. 126 min) and an overall shorter total ischemic time. No mortality was observed in this cohort. Therefore, public education campaigns must be initiated to increase public knowledge of the signs of acute coronary syndrome and the significance and advantages of calling for emergency ambulance services on 999.

The overall mortality rate for STEMI in our registry was 6.7%, with in-hospital mortality accounting for the majority (4.2%). Our in-hospital mortality rate remained lower than that of other registries, including the Global Registry of Acute Coronary Events (GRACE) at 7% and the Euro Heart Survey ACS II at 6% [9]. Moreover, the mortality rate after one year was comparable to that in affluent European countries. The Denmark Heart Registry, which has treated all patients with STEMI with PPCI for the past 20 years, has recorded a one-year mortality rate of 7.7%. Furthermore, based on the US Nationwide Emergency Departments Cohort Study, 7.5% of patients with STEMI in the United States died during the first year after PPCI [10]. The first year of the BaSSaL program has been both challenging and rewarding, as we continue along the path towards better cardiac patient care in Bahrain.

Limitations

This study had some limitations. First, despite the large number of patients, 60% were not citizens of Bahrain, which affected the demographic data. Most of these patients were young males with low socioeconomic status (blue-collar workers with limited access to healthcare follow-up and guideline-directed medical treatment after PPCI), which may affect adherence to treatment and the choice of antiplatelet therapy (i.e., despite the guidelines’ recommendation against using clopidogrel after PPCI to reduce the risk of stent failure, physicians chose to use it frequently because it is less expensive than ticagrelor and prasugrel, although it is not recommended unless ticagrelor/prasugrel are not available, not tolerated, or otherwise contraindicated). Moreover, these low-income patients are unlikely to be able to afford staged PCI for complete revascularization, even with strong evidence of survival benefits; therefore, the study outcomes must be classified based on nationality. The BaSSaL program demonstrates the urgent need for significant adjustments to healthcare policy, such as the implementation of national health insurance for all employees, including foreign workers. Life-saving treatments, such as reperfusion therapy for patients undergoing PPCI and staged revascularization, as well as guideline-directed medical therapy for coronary artery disease, should be covered by health insurance. Second, because our study only included individuals with acute STEMI, our findings do not apply to other patients with ACS.

## Conclusions

Nationwide quality control registries for patients with STEMI, such as the BaSSaL initiative, identify specific challenges in guideline adherence and delineate actions to ensure that the majority of STEMI patients receive lifesaving PPCI. Improving access to PPCI will reduce total ischemic time and mortality and help achieve target revascularization times.
